# Functional outcome of total knee replacement: a study protocol for a prospective, double-blinded, parallel-group randomized, clinical controlled trial of novel, personalized and conventional implants

**DOI:** 10.1186/s12891-019-2830-7

**Published:** 2019-10-12

**Authors:** T. Irmola, J. Kangas, A. Eskelinen, M. Niemeläinen, H. Huhtala, V. M. Mattila, T. Moilanen

**Affiliations:** 1 Coxa Hospital for Joint Replacement, Tampere, Finland; 2 Tampere University, Tampere, Finland; 3 Department of Orthopaedics and Trauma, Tampere University Hospital, Tampere, Finland

**Keywords:** Knee osteoarthritis, Arthroplasty, Total knee replacement, Novel designs, Functional outcome, Prospective studies, Clinical protocols

## Abstract

**Background:**

The development of total knee replacement (TKR) implant designs aims to improve outcome regarding pain, function, joint stiffness, instability, patellar problems, and ultimately wear of the implant. Recently, two major orthopaedic implant manufacturers launched a new generation of TKR implants which, according to the manufacturers, provide improved functional outcome. However, the benefits of these new TKR designs claimed by the manufacturers in terms of improved functional outcome still lack scientific documentation. The present randomized controlled trial has been designed to compare three fixed bearing, cemented cruciate-retaining (CR) designs; one of the new personalized TKR design with two conventional TKR designs with the main emphasis being on functional outcome.

**Methods:**

The present study is a prospective, double-blinded, randomized, single-center intervention trial. A total of 240 patients will be recruited to participate in a parallel-group study at Coxa Hospital for Joint Replacement, Tampere, Finland. We will compare the short-term functional outcome of TKR performed with a novel personalized TKR design (Persona CR, Zimmer, Warsaw, IN, USA) against TKRs performed with two conventional designs (PFC CR, DePuy, Warsaw, IN, USA and Nexgen CR, Zimmer, Warsaw, IN, USA). In total, 80 patients will be randomized in each of the three study arms. The primary outcome in this study is the Oxford Knee Score (OKS), which is a validated patient-reported outcome measure (PROM). Secondary outcome measures include the Forgotten Joint Score, the 15D, the UCLA activity score, and the VAS pain scale. The results will be analyzed after 2-year follow-up.

**Discussion:**

This paper presents a prospective, randomized, single-center trial study protocol. It provides details of patient randomization, PROMs, follow-up, methods of analysis of the material, and publication plan. An important aspect that will be considered in the study will be the economic effects of the novel designs as they are substantially more expensive, and the benefits of the added costs remain unknown. In addition, it is especially important to carry out evaluative studies in independent centers that are not biased by the interests of the manufacturers.

**Trial registration:**

Retrospectively registered, November, 2017, ClinicalTrials.gov Identifier: NCT03339557.

## Background

Total knee replacement operations are well-documented and effective treatments to decrease pain and improve function in patients with end-stage arthritis of the knee joint [1–3]. Moreover, knee replacements have been shown to be effective both clinically and in terms of cost effectiveness [4, 5]. Demand for joint replacement operations has continuously increased, and future projections suggest that this trend will continue [6], which will pose increasing demands on health care systems.

Although joint replacement surgery is widely considered to be one of the success stories of modern medicine, approximately up to 20% of patients with a knee replacement are to some extent dissatisfied with the outcome following their joint replacement operation [7–9].

### Evaluation of treatment

Measuring the outcome of total knee replacement surgery is challenging. Earlier, implant survival, i.e., the absence of revision surgery, was the most referenced measure of success. With contemporary implants and surgical techniques, the survival of most knee implants has been shown to be excellent even in long-term follow-up, as reported by many national joint registries [10–14]. Moreover, most implant designs have cumulative revision rates below 1% per annum, a target level set by the National Institute of Healthcare and Clinical Excellence (NICE) [15]. Recently, however, more emphasis has been put on patient satisfaction. Patient reported outcome measures scores (PROMs) have been introduced and used instead of traditional surgeon-based clinical assessments [16, 17]. One of the most widely used PROMs is the Oxford knee score (OKS), which is also included in the National Joint Registry for England and Wales data as an outcome measure [11, 18]. Other commonly used PROMS include the Visual Analogue Scale (VAS), the health-related quality of life (15D), and the UCLA activity score [19, 20]. At present, however, there is no single score or method for the assessment of all outcome aspects with widespread agreement, and newer and more sensitive scores, such as the “Forgotten joint score” (FJS), have been introduced to better capture the differences in function after TKR in more active patients [21–23].

### Previous studies

In addition to the patient related factors, surgical technique as well as the knee replacement design used influence the outcome [24] . Based on large registry data, it has been postulated that the effect of surgical factors, including implant type, are modest when compared with patient related factors [25].

The significance of implant design on knee function has been widely studied [26–29], as there is broad variability in implant concepts and specific designs. Numerous studies, however, have been unable to detect any differences in function between the most commonly used cruciate retaining (CR) or substituting, posterior stabilised (PS) concepts [26, 28]. Using the OKS as an outcome measure, one registry-based study showed that the performance of one implant type was statistically significantly better than all of the other most used contemporary implant designs [24]. However, the differences between the groups were not even close to the recently described minimally clinically important difference [30]. On the other hand, one small study showed that refining a well performing knee design did not influence the functional outcome [31] . In contrast to this finding, a new design was reported to improve the OKS score of TKR patients in a cross-sectional setting [32]. However, due to obvious limitations in study settings, these results must be interpreted with caution.

The effect of implant design on the outcome of knee replacement surgery is clearly being emphasized by the implant manufacturing industry. New, personalized and hopefully improved implant designs are the target of intensive research and development projects. A more anatomically accurate implant, finer sizing increments and full continuum of bearing constraints is redefining personalization. Recently, two major orthopaedic implant manufacturers both launched a new generation total knee replacement implant on to the market. Both of these implant systems are based on the legacy of their predecessors, which have been among the most implanted and best-documented designs globally. The clearly stated aim of these new systems is to improve functional outcome with less pain, joint stiffness, instability, patellar problems, and ultimately wear of the implant. However, the benefits claimed for these new or redesigned models in terms of improved outcome still lack scientific documentation. Cost is also an important aspect as the novel designs are substantially more expensive, and therefore the benefits of the added costs remain unknown. Furthermore, it is especially important to carry out evaluative studies in independent centers that are not biased by the interests of implant manufacturers.

### Study objective

The aim of this randomized controlled trial is to compare one novel, personalized TKR implant design (Persona CR, Zimmer, Warsaw, IN, USA) against two conventional TKR design (PFC CR,DePuy, Warsaw, IN, USA and NexGen CR, Zimmer, Warsaw, IN, USA) in terms of functional outcome and cost-effectiveness.

## Methods

The study design is a prospective, double-blinded randomized controlled, single-center trial adhering to Standard Protocol Items: Recommendation for Interventional Trials (SPIRIT) guidelines. The study will be carried out at Coxa Hospital for Joint Replacement (Tampere, Finland), a university-affiliated publicly-funded orthopaedic hospital specializing in joint replacement surgery. Currently, more than 4000 joint replacements are performed annually (representing approximately 20% of all hip and knee replacements performed annually in Finland), of which 2000 are primary knee replacement operations. Coxa Hospital is responsible for joint replacement surgery in Pirkanmaa Hospital District with a catchment area population of ca. 550,000 inhabitants, and it also serves as a tertiary referral hospital for one-fifth of Finland.

### Primary and secondary outcome measures

The primary outcome is the Finnish language version of the OKS (33) measured both preoperatively**,** and postoperatively at 2–3, 12, and 24 months. The OKS is a validated and reliable PROM. The questionnaire comprises 12 items regarding pain and activities of daily living (ADL) specifically related to the knee. Each item is scored on a five-level Likert scale 0 (worst disability) to 4 (no disability). The total score can range from 0 to 48, with 48 being the best possible score. The minimal clinically important difference (MCID) depends on the purpose of the comparison: it has recently been defined as 5 points, when different patient cohorts are compared against each other [18, 30, 33, 34]. Thus, in this study, a > 5-point difference in median OKS at 24 months postoperatively between any of the three cohorts studied (Persona CR, PFC CR, and NexGen CR) will be defined as a clinically significant difference in functional outcome.

The secondary measured outcomes both preoperatively, and after surgery at 2–3, 12, and 24 months are the FJS, the 15D, the UCLA activity score, and the VAS pain scale.

The FJS is an assessment tool designed especially for well-performing patients and describes the patients’ ability to forget the artificial joint in everyday life. The questionnaire comprises 12 items regarding and integrating pain, stiffness, function in ADL, patients’ activity levels, and psychosocial factors. Each item is scored on a five-point Likert response format of 1 (never) and 5 (mostly), and scores are summed and linearly transformed to a 0 to 100 scale. The high scores indicate good outcome, equaling a high degree of forgetting the replaced joint during ADL [17, 22, 35]. The validity and reliability of the FJS has been reported to be good, with a lower ceiling effect than the OKS [36].

The 15D is a generic, comprehensive (15-dimensional) self-administered instrument for measuring health-related quality of life (HRQoL)(19). The questionnaire comprises 15 dimensions with 5 ordinal levels on each dimension. From each dimension, the respondents choose the level that best describes their present health status. A set of population-based preference and utility weighs is used to generate the 15D score on a 0 (being dead) to 1 (full health) scale. Moreover, the 15D scores are generalizable and valid for deriving quality-adjusted life years (QALYs). The generic MCID for the change of 15D scores are ±0.15 [19, 37].

The 10-point University of California Los Angeles (UCLA) activity score has 10 descriptive activity levels ranging from wholly inactive (level 1) to regular participation in impact sports (level 10) [20].

The visual analogue scale (VAS) pain score, comprising a scale from 0 (no pain) to 10 (worst imaginable pain), is used to evaluate pain. In cost-effectiveness analysis, cost of surgery, hospitalization, out-patient visits and physiotherapy visits will be calculated. Cost per quality-adjusted life-years will be analyzed.

To further analyze the differences and complex relationships between pain experience and joint function, a sample of synovial tissue will be analyzed for patient specific factors associated with inflammation and inflammatory pain. These analyses will include immunohistology and measurements of gene expression and polymorphism relating to modulating pain and inflammatory reaction.

### Hypotheses

Our primary hypotheses in the study are the following:

i. The two conventional TKR implant designs and the novel implant design will yield similar functional outcomes measured with OKS
ii. Total knee replacement with either of the two conventional TKR implant designs will be more cost-effective than TKR with the novel implant design regarding quality-adjusted life years (QALYs) measured with the 15D
iii. High pain sensitivity will be associated with poor functional outcome irrespective of the implant design or radiographic severity of knee osteoarthritis (OA).

### Patient recruitment

**Fig. 1:**
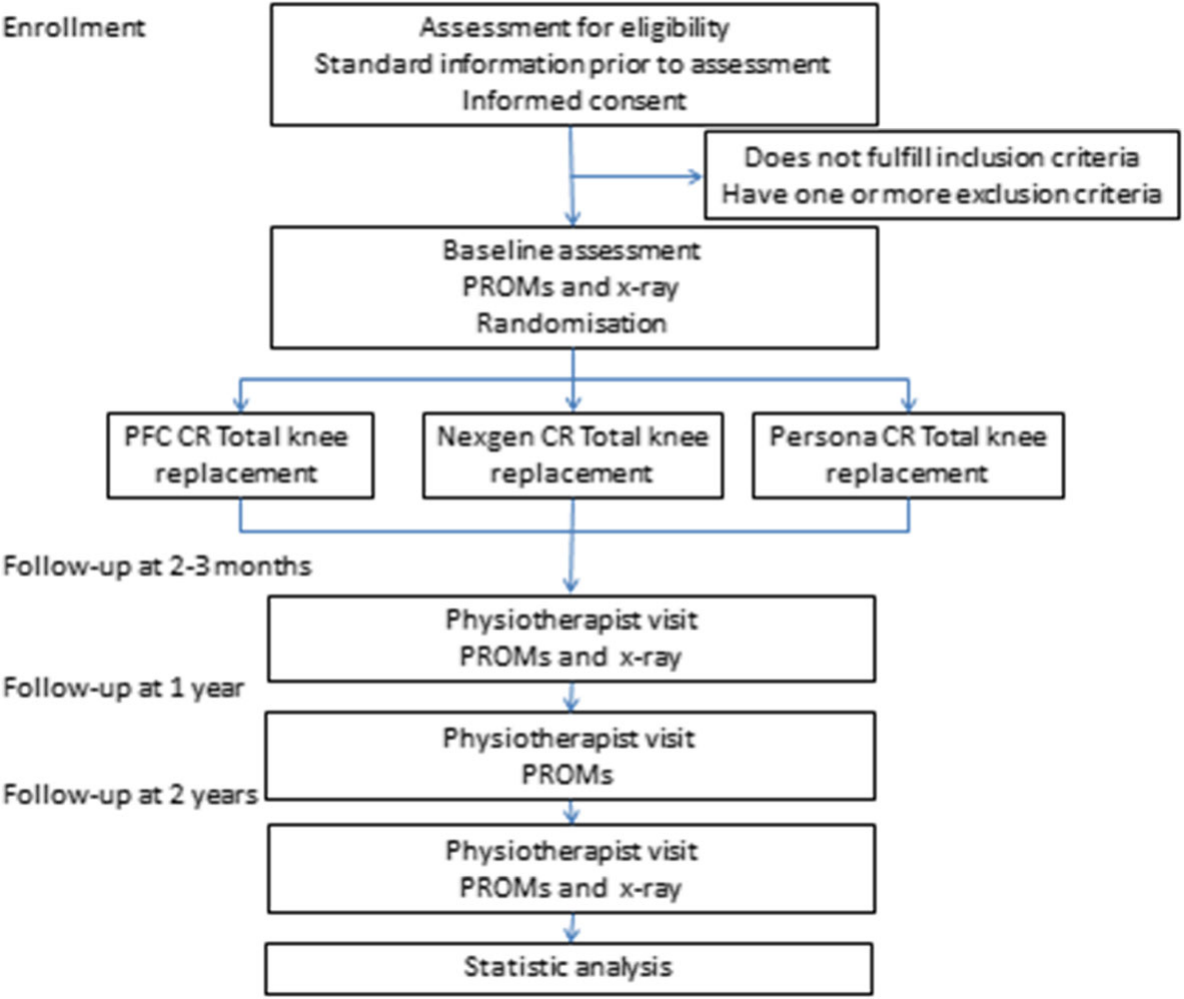
Patient flow through the trial (PROM: Patient Reported Outcome Measures)

Patients will be recruited from Coxa’s outpatient clinic according to our inclusion and exclusion criteria. The participating orthopaedic surgeons will recruit patients alongside their routine out-patient work. Written informed consent will be obtained. In the study, the indications for surgical treatment will follow the routine clinical guidelines of the hospital (Fig. 1).

### Patient inclusion criteria

- patients undergoing total knee replacement surgery for primary knee OA
- no previous open major surgery in the joint (e.g., osteotomy)
- age 50–70 years
- unilateral operative treatment with no plans for surgical treatment of the contralateral knee in the near future
- patients living in the local hospital district (Pirkanmaa Hospital District)
- Kellgren-Lawrence grade 3–4 knee osteoarthritis in plain radiographs

### Patient exclusion criteria

- unwilling to provide informed consent
- > 15 degrees varus or valgus, or > 15 degrees fixed flexion deformity
- predominantly patellofemoral osteoarthritis
- Physical, emotional, or neurological conditions that would compromise the patient’s compliance with postoperative rehabilitation and follow-up (e.g., drug or alcohol abuse, serious mental illness, general neurological conditions, such as Parkinson, MS, etc.)

### Patient randomization

When TKR surgery is decided, the eligible patients will be informed and those willing to participate will be randomized into one of the three implant groups. Block randomization lists will be computer-generated. Block-size will be variable with equal allocations to the three study groups. Numbered, sealed envelopes will be prepared. The trial coordinator will open the next (in number) sealed randomization envelope pre-operatively and reveal allocation. Opening of the envelope will be done after the surgeon has ascertained that the patient meets the eligibility criteria and that all of the implant types are feasible for the patient. The allocated implantation will then be performed, and the envelope, with study number indicated, returned to the trial coordinator.

### Preoperative patient evaluation

The preoperative medical history will be carefully documented according to routine screening for TKR surgery. The preoperative planning will include plain radiographs of the operated knee and standing long axis radiograph of the affected limb. Specific attention will be paid to record history of chronic pain, long- standing pain medication, a history of fibromyalgia, depression, anxiety, or other mental disturbances.

In addition to our normal preoperative protocol (including OKS), the patient will be asked to complete the following questionnaires/tests: the 15 D, the UCLA activity score, the FJS, and questionnaires for pain sensitivity and brief pain inventory.

### Operative treatment

Patients will be operated upon using the implant allocated in the randomization. Perioperative treatment will be carried out according to the routine protocol of the hospital: total knee arthroplasties (TKA) will be performed using the medial parapatellar approach, and the decision to use / not to use a tourniquet will be based on the surgeon’s preference. The mechanical alignment [38] technique will be used. The measured resection [39] technique, a combination of bony landmarks (femoral epicondyles, posterior condylar axis, and anterior-posterior axis), will be used to determine proper rotation of the femoral component. Distal femoral resections will be performed first using an intramedullary guide, which will be followed by a posterior referencing cutting block used to identify the proper component size. An appropriate 4-in-1 resection block will be used to perform anterior, posterior, and chamfer bone cuts in the femur. An extramedullary alignment guide will then be used to perform the tibial cut. Soft-tissue balance will be evaluated using either directly trial components or by first assessing ligamentous and gap balance with a tenser-device. Again, this will be based on the individual surgeon’s preferences. With trial components, range of motion, ligamentous balance, as well as extension-flexion gaps will be always assessed and recorded by the operating surgeon. If necessary, soft-tissue releases and ligament balancing will be performed to balance gap differences and/or varus/valgus ligamentous balance. All components implanted will be cemented, and the patella will only be resurfaced if there is a problem with patellar tracking. The TKAs will be carried out under spinal anesthesia in combination with intravenous sedation. General anesthesia will only be used if there is a contraindication to spinal anesthesia. Immediate full weight bearing will be allowed, and all patients will be mobilized on the day of the surgery. Antithrombotic prophylaxis, with low molecular-weight heparin, enoxaparin will be administered for 3 to 4 weeks postoperatively. All details of perioperative care and possible complications will be recorded in the hospital’s electronic database in a routine manner.

A 10 g sample of synovial tissue will be obtained during surgery. The study protocol will not require any additional tissue removal during surgery.

### Postoperative follow-up

The first postoperative visit to a physiotherapist blinded to the allocation will take place at 2 to 3 months after surgery. This visit will include a routine physical examination, plain radiographs of the operated joint, and the OKS score. In addition, the screening questionnaires/tests will be repeated, as described in Table 1. All complications and re-operations of the operated knee will be recorded.

**Table 1:** The schedule of enrollment, interventions, and assessments

	Study period					
	Enrollment	Allocation	Post-allocation			Close-out
Timepoint^a^	*-t*_*1*_	0	*t*_*1*_	*t*_*2*_	*t*_*3*_	*t*_*4*_
Enrollment						
Eligibility screen	X					
Informed consent	X					
Medical history	X					
Allocation		X				
Interventions:						
PFC CR Total knee replacement			X			
NexGen CR Total knee replacement			X			
Persona CR Total knee replacement			X			
Assessments:						
X-ray	X			X		
Pain sensitivity (PCS)	X					
Pain inventory (BPI)	X					
Oxford Knee Score (OKS)	X			X	X	X
15D	X			X	X	X
Forgotten Joint Score (FJS)	X			X	X	X
UCLA activity score	X			X	X	X
Surgeon visit	X					
Physiotherapist visit	X			X	X	X

^a^*-t*_*1*_ = baseline, *t*_*1*_ = operative treatment, *t*_*2*_ = postoperative at 2–3 months, *t*_*3*_ = at 1 year, *t*_*4*_ = at 2 years

The follow-up visits at 1 and 2-year intervals are additional to normal clinical practice. They will be performed by a physiotherapist blinded to the allocation and will include repeating the follow-up questionnaires, as shown in Table 1. All complications and re-operations of the studied knee will be recorded.

Further to the present work-flow, follow-up visits will be planned to take place at 5, 10, 13, and 16 years from the index operations to assess the mid- to long-term outcome and implant survivorship.

### Sample size

The power analyses will be calculated using both the OKS (primary outcome) and the FJS (secondary outcome). Of these, the FJS is more sensitive in differentiating patients at the upper end of the outcome scores and requires larger patient groups. With the OKS, the estimated sample size is 48 patients per arm. With the FJS in the range of 60 to 80 and a standard deviation of 25 points, the estimated sample size is 64 patients per arm (delta = 13, sd = 25, alpha = 0.05, power = 0.8). Allowing for a 10% drop-out rate and a 10% addition due to skewness in the variable distribution, the required arm size is 80. Therefore, with three comparison arms, the total number of patients recruited into the study will be 240.

### Blinding

Participants will be blinded to the implant design used in their operation. Staff on the ward will also be blinded. The physiotherapists conducting the follow-up visits (at 2–3 months, 1 year, and 2 years, i.e., the outcome assessors) will also be blinded to the allocation. The patients will not receive information on the specific implant design used in their operation until all patients have completed the 2-year follow-up visit.

### Statistical methods

Descriptive statistics, including mean and standard deviation, for continuous variables will be used. Differences between groups in continuous skewed main outcome variables will be analyzed using the Mann-Whitney U-test, and t-test when variables are normally distributed. Results will be presented with 95% confidence intervals. Two-way-tables with the chi-square test will be used for dichotomous variables, and multivariate analysis will be conducted with regression analysis. The α-level will be set at 0.05. SPSS Statistics version 23 (IBM Corp., Armonk, NY) will be used for the analysis.

### Study material

All the information and gathered material will be stored in a study registry at Coxa Hospital for Joint Replacement. The registry will be protected with passwords that will only be given to the authors and the secretary of the study group. All study data will be deleted 15 years after the end of the study, as required by Finnish law.

### Time schedule

Recruitment for the study began in September 2015 and was completed in August 2018. The results of the study will be analyzed after 2-year follow-up, and the final report will be published by the end of 2021.

## Discussion

This paper presents a prospective, randomized controlled trial comparing a novel TKR implant design with conventional TKR designs in terms of functional outcome and cost-effectiveness. It describes details of patient randomization, PROMs, follow-up, aftercare and methods of analysis of the material, and publication plan. The strengths of our study include its pragmatic nature and validated outcome measures, with an emphasis on PROMS. We included several aspects of outcome to capture the whole picture of patient satisfaction and to obtain data on the economic aspect for cost/benefit analyses.

The adequate number of patients is a very critical question. According to power analysis, our study design allows us to have sufficient numbers of patients to confirm or reject the research hypotheses on primary outcome measures. This is especially important if the results turn out to show no differences between the study arms – to avoid speculation on the possibility of a type 2 error. The economical aspect is also important as the novel designs are substantially more expensive, and therefore the benefits of the additional costs are of great interest to all health care providers.

Maintaining blinding of study arms is challenging in all studies that assess the outcomes of surgical procedures. Successful blinding of the patients is critical because PROMS are the key outcome tools. We discussed the nature and importance of blinding with the patients before they consented to participate in the study. The follow-up visits are being carried out by physiotherapists who are dedicated to research projects, and their perfect blinding is also challenging and depends largely on their commitment to the study philosophy.

Strict inclusion criteria are a possible limitation of our study and may result in selection bias, as a good number of patients had to be excluded. Therefore, even random allocation may end up in heterogeneity of intervention arms. However, the inclusion criteria are set to focus on the most common type of patient, i.e., patients with primary uncomplicated osteoarthritis of the knee. The upper age limit was set to 70 years to also obtain outcome data on the more active younger TKR patients, who tend to end up at the upper end of the outcome scores after TKR surgery. In theory, this approach might help us to better differentiate “good” outcome from an “excellent” outcome, especially if such subtle differences are found between the implant designs that would not become evident in the older, less active patient groups. On the other hand, if a novel knee design cannot provide these younger and generally more active patients with an improved functional outcome, it will certainly not do so in other patient groups either.

Introducing a new TKR implant system is a known risk factor for failure in joint replacement [40]. This poses the danger of a type 2 error in hypothesis 1, as the surgeons are more familiar with the conventional implant systems and the outcome of the novel TKR design may be inferior due to surgeon-related factors. To avoid this pitfall, we arranged a formal training of the novel implant system and allowed each of the participating surgeons to perform approximately 10 surgeries with the novel implant before commencing the random allocation.

The longevity of contemporary TKR designs is excellent, but the functional outcome and patient satisfaction levels still leave room for improvement. Functional outcome is, however, the result of complex interplay, where the implant design is only one factor and patient characteristics and the surgeon performing the implantation play major roles as well. The development of TKR implants is a continuous activity, where the manufacturing industry plays a leading role. The evaluation of novel designs must be carried out according to strict scientific methods to be able to differentiate the influence of the three major factors – the implant, the patient, and the surgical performance – from each other. In addition, it is especially important to carry out these evaluative studies in independent centers, and not to be biased by the interests of implant manufacturers.

## Data Availability

The datasets used and/or analyzed during the current study will be available from the corresponding author on reasonable request once the study has been completed.

## Abbreviations

ADL: Activities of daily living
CR: Cruciate retaining
FJS: Forgotten joint score
HRQoL: Health-related quality of life
MCID: Minimal clinically important difference
NICE: National Institute of Healthcare and Clinical Excellence
OA: Osteoarthritis
OKS: Oxford Knee Score
PROM: Patient-reported outcome measure
PS: Posterior stabilized
QALYs: Quality-adjusted life years
SPIRIT: Standard Protocol Items: Recommendation for Interventional Trials
TKA: Total knee arthroplasty
TKR: Total knee replacement
UCLA: University of California Los Angeles
VAS: Visual-Analogue-Scale

## References

[1] Ethgen O, Bruyere O, Richy F, Dardennes C, Reginster J (2004). Health-related quality of life in total hip and total knee arthroplasty: a qualitative and systematic review of the literature. J Bone Joint Surg.

[2] Skou ST, Roos EM, Laursen MB, Rathleff MS, Arendt-Nielsen L, Simonsen O (2015). A randomized controlled trial of total knee replacement. N Engl J Med.

[3] Skou ST, Roos EM, Laursen MB, Rathleff MS, Arendt-Nielsen L, Rasmussen S (2018). Total knee replacement and non-surgical treatment of knee osteoarthritis: 2-year outcome from two parallel randomized controlled trials. Osteoarthr Cartil.

[4] Räsänen P, Paavolainen P, Sintonen H, Koivisto A, Blom M, Ryynänen O (2007). Effectiveness of hip or knee replacement surgery in terms of quality-adjusted life years and costs. Acta Orthop.

[5] Jenkins PJ, Clement ND, Hamilton DF, Gaston P, Patton JT, Howie CR (2013). Predicting the cost-effectiveness of total hip and knee replacement: a health economic analysis. Bone Joint J.

[6] Kurtz S, Ong K, Lau E, Mowat F, Halpern M (2007). Projections of primary and revision hip and knee arthroplasty in the United States from 2005 to 2030. J Bone Joint Surg Am.

[7] Bourne R, Chesworth B, Davis A, Mahomed N, Charron K (2010). Patient satisfaction after total knee arthroplasty: who is satisfied and who is not?. Clin Orthop Relat Res.

[8] Scott CEH, Howie CR, MacDonald D, Biant LC (2010). Predicting dissatisfaction following total knee replacement: a prospective study of 1217 patients. J Bone Joint Surg.

[9] Beswick AD, Wylde V, Gooberman-Hill R, Blom A, Dieppe P (2012). What proportion of patients report long-term pain after total hip or knee replacement for osteoarthritis? A systematic review of prospective studies in unselected patients. BMJ Open.

[10] Aust Orthopaedic Assoc (2017). National Joint Replacement Registry.

[11] National Joint Registry fo England, Wales (2017). NJR 14th annual report 2017.

[12] Norwegian National Unit on Arthroplasty (2017). Nasjonalt register for Leddproteser annual report.

[13] New Zealand Orhtopedic Association (2017). The New Zealand joint registry.

[14] Robertsson O, Lidgren L, Sundberg M, W-Dahl A. The Swedish Knee Arthroplasty Register Annual Report 2017. 2017. 10.1302/2046-3758.37.2000289 PMC4112790 24986492

[15] Anonymous (2015). ODEP statement.

[16] Giesinger K, Hamilton DF, Jost B, Holzner B, Giesinger JM (2014). Comparative responsiveness of outcome measures for total knee arthroplasty osteoarthritis and cartilage / OARS. Osteoarthr Res Soc.

[17] Hamilton DF, Gaston P, Simpson AH (2012). Is patient reporting of physical function accurate following total knee replacement?. J Bone Joint Surg.

[18] Dawson J, Fitzpatrick R, Murray D, Carr A (1998). Questionnaire on the perceptions of patients about total knee replacement. J Bone Joint Surg.

[19] Sintonen H (2001). The 15D instrument of health-related quality of life: properties and applications. Ann Med.

[20] Zahiri CA, Schmalzried TP, Szuszczewicz ES, Amstutz HC (1998). Assessing activity in joint replacement patients. J Arthroplasty.

[21] Behrend H, Giesinger K, Giesinger JM, Kuster MS (2012). The "forgotten joint" as the ultimate goal in joint arthroplasty. J Arthroplasty.

[22] Thienpont E, Opsomer G, Koninckx A, Houssiau F (2014). Joint awareness in different types of knee arthroplasty evaluated with the forgotten joint score. J Arthroplasty.

[23] Klit J, Jacobsen S, Rosenlund S, Sonne-Holm S, Troelsen A (2014). Total knee arthroplasty in younger patients evaluated by alternative outcome measures. J Arthroplasty.

[24] Baker PN, Deehan DJ, Lees D, Jameson S, Avery PJ, Gregg PJ (2012). The effect of surgical factors on early patient-reported outcome measures (PROMS) following total knee replacement. J Bone Joint Surg.

[25] Keurentjes JC, Fiocco M, So-Osman C, Onstenk R, Gemert K-V, Ankie WMM (2013). Patients with severe radiographic osteoarthritis have a better prognosis in physical functioning after hip and knee replacement: a cohort-study. PLoS One.

[26] Berend KR, Lombardi J, Adams JB (2013). Which total knee replacement implant should I pick? Correcting the pathology: the role of knee bearing designs. Bone Joint J.

[27] van der Voort P, Pijls BG, Nouta KA, Valstar ER, Jacobs WCH, Nelissen RG (2013). A systematic review and meta-regression of mobile-bearing versus fixed-bearing total knee replacement in 41 studies. Bone Joint J.

[28] Becker R, Hirschmann M, Karlsson J (2014). Does implant design and surgical technique improve the clinical outcome in total knee arthroplasty?. Knee Surg Sports Traumatol Arthrosc.

[29] Nunley R, Nam D, Berend K, Lombardi A, Dennis D, Della Valle C (2015). New Total knee arthroplasty designs: do young patients notice?. Clin Orthop Relat Res.

[30] Beard DJ, Harris K, Dawson J, Doll H, Murray DW, Carr AJ (2015). Meaningful changes for the Oxford hip and knee scores after joint replacement surgery. J Clin Epidemiol.

[31] Piepers MJ, van Hove RP, van den Bekerom MPJ, Nolte PA (2014). Do refinements to original designs improve outcome of total knee replacement? A retrospective cohort study. J Orthop Surg Res.

[32] Thomsen MG, Latifi R, Kallemose T, Husted H, Troelsen A (2016). Does knee awareness differ between different knee arthroplasty prostheses? A matched, case-control, cross-sectional study. BMC Musculoskelet Disord.

[33] Reito A, Jarvisto A, Jamsen E, Skytta E, Remes V, Huhtala H, et al. Translation and validation of the 12-item Oxford knee score for use in Finland. BMC Musculoskelet Disord. 2017;18. 10.1186/s12891-017-1405-8. 10.1186/s12891-017-1405-8 PMC5299663 28178956

[34] Murray DW, Fitzpatrick R, Rogers K, Pandit H, Beard DJ, Carr AJ (2007). The use of the Oxford hip and knee scores. J Bone Joint Surg.

[35] Behrend H, Zdravkovic V, Giesinger J, Giesinger K (2016). Factors predicting the forgotten joint score after Total knee arthroplasty. J Arthroplasty.

[36] Thomsen MG, Latifi R, Kallemose T, Barfod KW, Husted H, Troelsen A (2016). Good validity and reliability of the forgotten joint score in evaluating the outcome of total knee arthroplasty. Acta Orthop.

[37] Alanne S, Roine RP, Räsänen P, Vainiola T, Sintonen H (2015). Estimating the minimum important change in the 15D scores. Qual Life Res.

[38] Insall JN, Binazzi R, Soudry M, Mestriner LA. Total knee arthroplasty. Clin Orthop Relat Res. 1985;(192):13–22. 3967412

[39] Hungerford DS, Krackow KA (1985). Total joint arthroplasty of the knee. Clin Orthop Relat Res.

[40] Peltola M, Malmivaara A, Paavola M (2012). Introducing a knee Endoprosthesis model increases risk of early revision surgery. Clin Orthop Relat Res.

